# Toothache

**Published:** 1884-04

**Authors:** 


### TOOTHACHE.

"It is a significant fact that toothache is almost exclusively an affliction of the northern nation."—*Felix L. Oswald, M. D., in Pop. Sci. Monthly.*

We are accustomed to note all manner of scientific inaccuracies and misstatements in the daily press. But when so accomplished a writer as Dr. Oswald, in the leading journal of its class in this country, makes such an unqualified affirmation as this, some one should ask upon what authority it is done. It has been conclusively demonstrated that dental caries is more or less common to every nation; not only to these now in existence, but to the extinct races as well. Climatic influences and dietary habits only to a moderate extent serve to modify the ravages of this disease. Individual development and bodily conditions may resist it, as they do other morbid states, but that the inhabitants of any zone are free from dental diseases, and from the pain which necessarily accompanies them, should not be asserted by any medical man, unless upon undisputable testimony.

